# A Temporal Order in 5′- and 3′- Processing of Eukaryotic tRNA^His^

**DOI:** 10.3390/ijms20061384

**Published:** 2019-03-19

**Authors:** Marie-Theres Pöhler, Tracy M. Roach, Heike Betat, Jane E. Jackman, Mario Mörl

**Affiliations:** 1 Institute for Biochemistry, Leipzig University, Brüderstraße 34, 04103 Leipzig, Germany; marie.poehler@uni-leipzig.de (M.-T.P.); heike.betat@uni-leipzig.de (H.B.); 2 Center for RNA Biology and Ohio State Biochemistry Program, Department of Chemistry and Biochemistry, the Ohio State University, Columbus, OH 43210, USA; roach.211@buckeyemail.osu.edu (T.M.R.); jackman.14@osu.edu (J.E.J.)

**Keywords:** CCA-addition, G-1 residue, tRNA^His^ guanylyltransferase, tRNA^His^, tRNA maturation, tRNA nucleotidyltransferase

## Abstract

For flawless translation of mRNA sequence into protein, tRNAs must undergo a series of essential maturation steps to be properly recognized and aminoacylated by aminoacyl-tRNA synthetase, and subsequently utilized by the ribosome. While all tRNAs carry a 3′-terminal CCA sequence that includes the site of aminoacylation, the additional 5′-G-1 position is a unique feature of most histidine tRNA species, serving as an identity element for the corresponding synthetase. In eukaryotes including yeast, both 3′-CCA and 5′-G-1 are added post-transcriptionally by tRNA nucleotidyltransferase and tRNA^His^ guanylyltransferase, respectively. Hence, it is possible that these two cytosolic enzymes compete for the same tRNA. Here, we investigate substrate preferences associated with CCA and G-1-addition to yeast cytosolic tRNA^His^, which might result in a temporal order to these important processing events. We show that tRNA nucleotidyltransferase accepts tRNA^His^ transcripts independent of the presence of G-1; however, tRNA^His^ guanylyltransferase clearly prefers a substrate carrying a CCA terminus. Although many tRNA maturation steps can occur in a rather random order, our data demonstrate a likely pathway where CCA-addition precedes G-1 incorporation in *S. cerevisiae*. Evidently, the 3′-CCA triplet and a discriminator position A73 act as positive elements for G-1 incorporation, ensuring the fidelity of G-1 addition.

## 1. Introduction

Transfer RNAs (tRNAs) are essential key players in life. They function as adapter molecules, establishing an interface between the encoded genetic information in mRNA and the amino acid sequence at the protein level. To accomplish this critical biological function, tRNA transcripts undergo a large number of processing events in order to yield the mature tRNA species that are utilized by the translation machinery. Apart from the removal of 5′-leader, 3′-trailer and intron sequences (if present), the introduction of modifications at certain nucleotides and nucleotide editing are key maturation events [1]. Furthermore, all tRNAs carry the conserved CCA triplet at their 3′-end, generating the site of aminoacylation. While in some Bacteria and Archaea the CCA sequence is encoded in the tRNA genes, in all remaining organisms, especially in Eukarya, it is added post-transcriptionally by tRNA nucleotidyltransferase (CCA-adding enzyme) [2,3,4]. Nevertheless, CCA-adding enzymes occur in each domain of life, and according to their variable structural features and different mechanisms, they are divided into class I (Archaea) and class II (Bacteria and Eukarya) [5]. In class I CCA-adding enzymes, a highly conserved arginine residue in the active site and the sugar-phosphate-backbone of the tRNA acceptor stem collaborate in nucleotide selection and form hydrogen bonds with the incoming NTP [6], whereas in class II a pure amino acid based template exists. Here, the nucleotide binding pocket contains three highly conserved amino acids, forming Watson Crick-like hydrogen bonds to either CTP or ATP, without interacting with the tRNA [7]. Despite these differences, both types of CCA-adding enzymes incorporate the nucleotides at high fidelity to the tRNA 3′-end. Furthermore, they also play an important role in the quality control of tRNA transcripts [8,9,10,11].

Whereas the 3′-CCA-end is an indispensable feature for every mature tRNA, the addition of a single guanosine nucleotide at the tRNA 5′-end is a special maturation event occurring on tRNA^His^. The presence of this single G at the -1 position is critical for recognition of tRNA^His^ by the histidyl tRNA synthetase (HisRS) in nearly all bacteria, as well as in a large number of eukaryotes, with only a few notable exceptions described so far in each domain [12,13]. While these bacterial and eukaryotic tRNA^His^ species share the same G-1 element, the mechanisms of incorporation vary between the two domains. In Bacteria, the genomically encoded G-1 is retained after 5′-end cleavage of the tRNA^His^ precursor transcript due to an alternative cleavage pattern in the 5′-leader sequence exhibited by Ribonuclease P (RNase P) [14]. In Eukarya, however, G-1 is not encoded and must be added post-transcriptionally by tRNA^His^ guanylyltransferase (Thg1) [15,16]. Thg1 adds a single G-1 residue across from an A73 discriminator nucleotide, forming an unconventional non-Watson Crick base pair. This unusual 3′-5′ nucleotidyltransferase protein is a member of a larger family of Watson Crick-dependent 3′-5′ polymerase enzymes that share structural similarity with canonical 5′-3′ DNA and RNA polymerases [16].

The fact that tRNA^His^ is subject to post-transcriptional addition of 5′- as well as 3′-terminal features by highly conserved polymerases that act on either end of a partially matured precursor raises important questions about coordination between these enzymatic activities. Indeed, prior to the activities of Thg1 and CCA-adding enzyme, the precursor tRNA^His^ species, like all other pre-tRNA, is transcribed in the nucleus with additional 5′-leader and 3′-trailer sequences that are removed by the nuclear enzymes RNase P and ribonuclease Z (RNase Z), respectively [1]. This 5′- and 3′-end processed tRNA is subsequently exported to the cytoplasm for additional maturation events, including the activities of Thg1 and CCA-adding enzyme, which are both localized to the cytoplasm in *S. cerevisiae*. Although both enzymes share the tRNA acceptor stem as a substrate, there is no evidence for interaction between Thg1 and the CCA-adding enzyme [17]. Therefore, the possibility of competition between these 5′- and 3′-end maturation activities for the same end-matured tRNA^His^ substrate is raised, and whether there is any apparent order associated with these enzymatic events has not been investigated.

**Figure 1:**
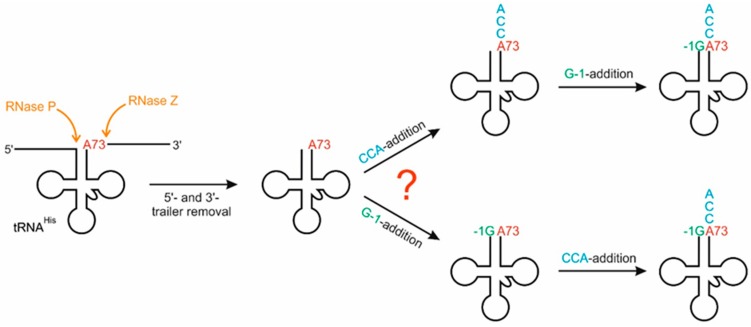
Cytosolic tRNA^His^ processing in eukaryotes. After removal of 5′-leader and 3′-trailer sequences by the nucleus-localized RNase P and RNase Z (orange), CCA- (cyan) and G-1-addition (green) take place in the cytosol. However, it is not clear whether these events occur at random or follow a sequential order, due to different substrate preferences of the involved enzymes. Furthermore, these events can be affected by the nature of the discriminator base 73 that is located across the -1 position (as an example, A73 is indicated in red).

Here we sought to investigate the order of tRNA^His^ processing on the 5′- and 3′-ends of tRNA^His^ in *S. cerevisiae* using recombinant enzymes and assessing the in vitro substrate preferences for each enzyme for the matured vs. precursor form with respect to the other enzyme. By determining the biologically relevant and preferred substrate for each enzyme, we aimed to understand enzyme recognition and patterns of activity (Figure 1). Knowing that tRNA^His^ as well as CCA-adding enzyme and Thg1 are localized in the cytosol of *S. cerevisiae*, we identified a sequential order of tRNA^His^ processing. In this scenario, according to our results the enzymes do not compete for the substrate. Instead, the differing substrate preferences lead to a sequential order of nucleotide incorporation at 5′- and 3′-ends, resulting in a mature tRNA^His^ in the cytosol.

## 2. Results

### 2.1. Eukaryotic Cytosolic tRNA^His^ Processing—A Temporal Order of 5′- and 3′- Nucleotide Incorporation

#### 2.1.1. Addition of the 3′-terminal CCA Sequence

**Figure 2:**
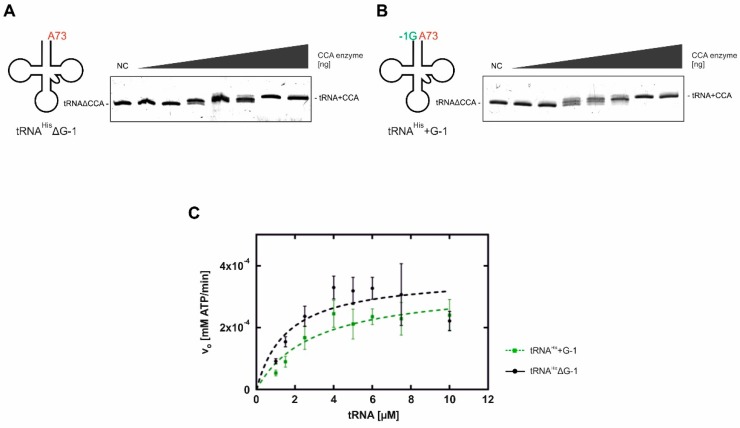
CCA-adding enzyme-catalyzed CCA incorporation on tRNA^His^ lacking G-1 (tRNA^His^ΔG-1; A) and tRNA^His^+G-1 (B) from *S. cerevisiae*. tRNA variants were incubated with increasing amounts of CCA-adding enzyme (0.5, 1.5, 3.0, 4.5, 6.0, 60 und 300 ng). The reaction products were separated on a denaturing polyacrylamide gel and visualized by ethidium bromide staining. Both tRNA variants were processed at comparable efficiencies, resulting in a complete substrate turnover. NC, negative control without enzyme. (C) Kinetic analysis of CCA-addition for both tRNA variants. Increasing amounts of tRNA^His^ΔG-1 (black curve) and tRNA^His^+G-1 (green curve) were incubated with CCA-adding enzyme, NTPs and α-^32^P-ATP under steady-state conditions. Michaelis–Menten data were calculated from triplicates using GraphPadPrism software (Table 1).

To figure out whether the maturation status of the 5′-end has an impact on the efficiency of CCA-addition at the 3′-end, in vitro transcribed cytosolic *S. cerevisiae* tRNA^His^ΔG-1 and tRNA^His^+G-1 were incubated with increasing amounts of recombinant CCA-adding enzyme. Reaction products were separated on denaturing polyacrylamide gels and visualized by ethidium bromide staining. In the resulting band patterns of the reaction products, no remarkable differences in the efficiency of CCA-addition on these substrates were observed (Figure 2A,B). Therefore, the CCA-adding enzyme is able to act on both substrates and efficiently incorporates the complete CCA triplet at the 3′-end. To further quantify these results, steady-state Michaelis–Menten kinetics for the complete CCA-addition were performed (Figure 2C). As the limited solubility properties of RNA do not allow for using excessive saturating conditions in these analyses, the obtained parameters represent apparent values typical for CCA-addition kinetics [18,19,20,21]. For both transcripts, we obtained similar apparent K_M_ values, indicating that the CCA-adding enzyme binds these two substrates with similar affinity. Furthermore, for both substrates tRNA^His^ΔG-1 and tRNA^His^+G-1, comparable turnover numbers (apparent k_cat_) with values of approximately 25 min^−1^ were determined (Table 1). Hence, these data indicate that the 3′-terminal CCA-addition is not affected by the processing status at the 5′-end of tRNA^His^.

#### 2.1.2. Incorporation of the G-1 Nucleotide

**Figure 3:**
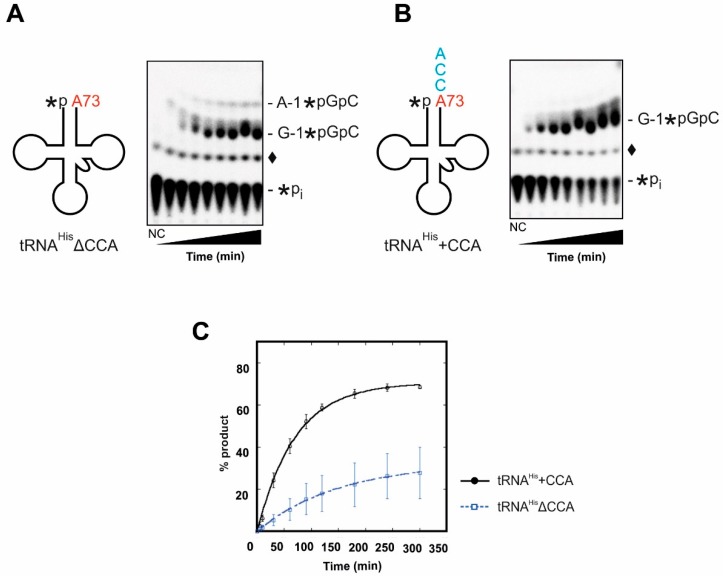
The CCA triplet affects the fidelity of G-1-addition by Thg1. 5′-labeled tRNA^His^ lacking the CCA-end (tRNA^His^ΔCCA); (**A**) or ending with CCA (tRNA^His^+CCA); (**B**) were incubated with saturating amounts of Thg1 (15 µM) and assayed using a phosphatase protection assay, in which the 5′-^32^P-label on unreacted tRNA substrate is accessible to phosphatase, and visualized as inorganic phosphate (*Pi). On tRNA^His^ΔCCA, Thg1 exhibits a reduced fidelity and adds not only the correct G-1 (G-1*pGpC product) but to a certain amount also erroneously A-1 (A-1*pGpC product on panel A). However, when the tRNA substrate carries a 3′-terminal CCA sequence, Thg1 exclusively incorporates G-1, indicating that the CCA triplet contributes to the fidelity of the reaction. An additional non-enzymatic labeled species (♦) is visible in the no enzyme control (NC) and enzyme-containing reactions, as has been observed previously with these types of labeled tRNA assays [13,23]. (**C**) Single-turnover nucleotide incorporation was measured in triplicate and plotted as a function of time. 5′-labeled tRNA^His^ΔCCA (blue) and tRNA^His^+CCA (black) were incubated with 15 µM Thg1 in the presence of 0.1 mM ATP and 1 mM GTP.

In a complementary series of experiments, we determined whether recombinant Thg1 prefers to act on tRNA^His^ before or after 3′-terminal CCA-addition. The unusual 3′-5′ nucleotide transfer mediated by Thg1 family proteins occurs in a 3-step reaction [22]. First, the 5′-monophosphate end of the substrate tRNA is adenylated via a 5′-5′-linkage with ATP as a co-substrate. Next, the 3′-hydroxyl of the incoming nucleotide (GTP) attacks the α-phosphate of the activated tRNA 5′-end, resulting in a phosphodiester bond. Lastly, pyrophosphate is released from the incorporated G-1 nucleotide. In order to allow this mechanism to proceed, the in vitro transcribed tRNA substrates with (tRNA^His^+CCA) and without 3′-CCA-end (tRNA^His^ΔCCA) were monophosphorylated at the 5′-end using γ-^32^P-ATP. Thg1-catalyzed nucleotide incorporation on tRNA^His^ΔCCA and tRNA^His^+CCA was monitored using a phosphatase protection assay which results in the protection of a labelled oligonucleotide (G-1*pGpC) from phosphatase activity if 5′-nucleotides have been incorporated (Figure 3A,B) [15]. As indicated by the intensity of the radioactive signals after resolution by TLC, Thg1 readily incorporates the G-1 nucleotide at the 5′-end of tRNA^His^+CCA (Figure 3B). On the substrate lacking the CCA-end (tRNA^His^ΔCCA), however, the enzyme is less efficient. Furthermore, Thg1 forms an additional minor product on tRNA^His^ΔCCA, which resolves higher than the expected G-1 spot (Figure 3A). RNase T1 treatment and resolution by PEI cellulose TLC identified this spot as a side-reaction product of A-1 addition (A-1*pGpG) (Figure S2). It appears that when tRNA^His^ lacks the 3′-CCA-end, Thg1 exhibits a certain infidelity by incorporating some incorrect nucleotides at the -1 position.

To address the differences in reaction efficiency on tRNA^His^ΔCCA compared to tRNA^His^+CCA in more detail, G-1 addition was performed under single-turnover conditions ([E]>>[S]; E, enzyme; S, substrate) to obtain k_obs_ values for each substrate. Although G-1 addition is clearly observed for both substrates, the measured k_obs_ for nucleotide incorporation by Thg1 in the presence of tRNA^His^+CCA is 2 times faster than that of tRNA^His^ΔCCA, supporting the qualitative observation in the TLC assays (Figure 3, Table 2). Furthermore, in addition to the effect on the rate of G-1 addition, the maximal amount of product formed in the absence of CCA was also significantly lower than in the presence of the intact 3′-end (Figure 3C). Finally, we obtained rates of nucleotide incorporation under varying enzyme concentrations, which enables the measurement of apparent dissociation constants (K_D,app_) for each tRNA substrate. A value of 5.8 ± 4.3 µM was obtained for tRNA^His^+CCA (Table 1). This value aligned well with previously published data [15]. In comparison, a precise dissociation constant for tRNA^His^ΔCCA was not attainable due to the inability to saturate the observed rate even at the highest concentration of protein achievable in our assays, and therefore leading us to estimate that K_D,app_ for tRNA^His^ΔCCA is ≥30 μM. Taken together, these results clearly indicate that tRNA^His^+CCA is a better substrate for Thg1 than tRNA^His^ΔCCA in terms of nucleotide incorporation rates, fidelity, and binding.

### 2.2. The 3′- A73CCA Sequence Serves as a Fidelity Determinant for Thg1

In contrast to almost all bacterial and archaeal tRNA^His^ species, which encode a cytosine residue at the discriminator position (C73), the eukaryotic cytosolic tRNA^His^ shows a conserved adenosine at position 73. Mutated tRNA^His^ variants that contain a C73 have been shown to affect the activity of Thg1, leading to the incorporation of multiple G residues onto the 5′-end of the tRNA, essentially “zipping” up the acceptor stem [24,25]. Since this 3′-5′ polymerase activity utilizes the 3′-CCA sequence as a template, the simultaneous presence of G-1 and CCA-end might lead to different consequences in the context of a C73 discriminator nucleotide instead of A73. We therefore tested whether the identity of the discriminator base has an impact on the correct incorporation of either the 3′-CCA sequence or the 5′-G-1 position. *S. cerevisiae* tRNA^His^ transcripts with and without G-1 were generated, carrying a C73 discriminator instead of the wild type A73 position (tRNA^His^ΔG-1 A73C and tRNA^His^+G-1 A73C). Due to the additional base pair G-1/C73, tRNA^His^+G-1 A73C carries an extended acceptor stem with a base-paired discriminator position (Figure S1B). However, this situation does not affect CCA-addition catalyzed by the CCA-adding enzyme and results in a similarly efficient CCA incorporation to tRNAHis+G-1 A73C compared to tRNA^His^ΔG-1 A73C (Figure S1A). Taken together, both tRNA^His^ substrates with a cytosine at the discriminator position are readily accepted as substrates for CCA-addition, showing comparable band patterns like the wild type tRNA^His^ containing an A73. Due to this identical substrate acceptance of the CCA-adding enzyme, no kinetic parameters were further determined.

**Figure 4:**
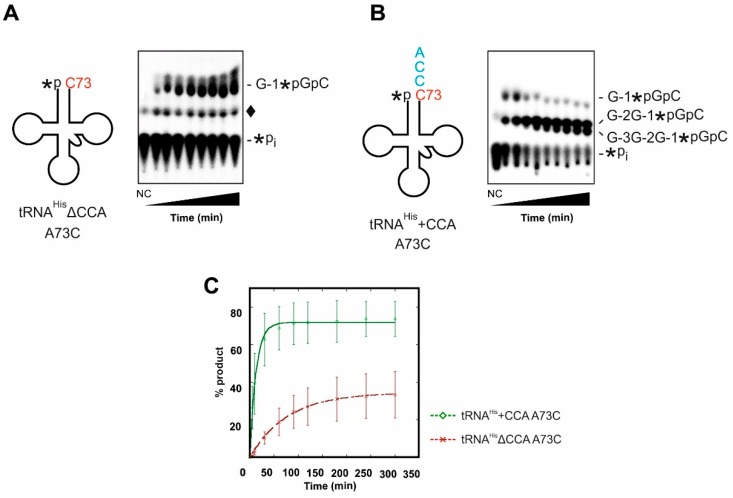
Thg1 activity on cytosolic tRNA^His^ carrying a cytosine residue at the discriminator position 73 (red). (**A**) On tRNA^His^ΔCCA A73C, the enzyme correctly adds a single G-1 residue (G-1*pGpC product), consistent with the absence of a 3′-end template sequence for further 3′-5′ polymerization. Other species observed in this assay include *Pi, which represents the remaining unreacted substrate tRNA, and ♦ which represents the non-enzymatic product that is visible in both no enzyme control (NC) and enzyme-containing reaction lanes. (**B**) If tRNA^His^ A73C additionally carries the 3′-CCA-end (cyan), Thg1 catalyzes multiple GTP incorporations in a 5′-template-dependent manner, resulting in two additional G-C base pairs with the 3′-CCA-end (G-2G-1*pGpC and G-3G-2G-1*pGpC products). In this case, the non-enzymatic side product is not visible because it is obscured by the strong signal from the multiple G-addition products. (**C**) Single-turnover nucleotide incorporation was measured in triplicate and plotted as a function of time. 5′-labeled tRNA^His^ΔCCA A73C (red graph) or tRNA^His^+CCA A73C (green graph) were incubated with saturating amounts of enzyme (15 µM), 0.1 mM ATP and 1 mM GTP.

Next, G-1 incorporation on tRNA^His^ A73C variants with and without 3′-CCA-end was investigated. As shown for the tRNA^His^ with A73, the time course experiments indicate a significant preference of Thg1 for tRNA^His^ containing the 3′-CCA, both in terms of rate and maximal amount of product formed in the reactions (Figure 4). In the kinetic analysis, the rates of nucleotide incorporation (0.097 min^−1^ for tRNA^His^+CCA A73C and 0.013 min^−1^ for tRNA^His^ΔCCA A73C) differ by nearly 10-fold (Figure 4C, Table 2). Finally, the substantial decrease in affinity for the tRNA in the absence of the 3′-CCA sequence was also observed here, as judged by the difference in K_D,app_ measured by single-turnover assay (Table 2). Interestingly, in the presence of the 3′-CCA-end, Thg1 incorporated multiple (up to 3) G nucleotides at the tRNA 5′-end, as the 3′-CCA sequence on a tRNA^His^ with C73 obviously acts as a template for incorporation of multiple nucleotides as described by Jackman and Phizicky (compare Figure 4A vs. Figure 4B) [25].

## 3. Discussion

To participate in protein biosynthesis, tRNAs undergo many different maturation steps. Differences in subcellular localization of various components of the tRNA maturation machinery enforces a temporal order to many of these modification events, in which the tRNA substrate’s movement around the cell after transcription dictates the next steps in terms of its lifecycle [26]. In contrast, processing events that occur in the same compartment have been much less well described, and whether there are substrate specificity preferences that lead to sequential processing activities or whether these activities are more random in nature has not been investigated in many cases. The case of histidine tRNA provides a unique and perfect opportunity to investigate this question, since the addition of two indispensable features- the 3′-CCA-end and the 5′-G-1 position- are both added post-transcriptionally by conserved enzymes that are found in the cytosol of *S. cerevisiae*. Hence, it is conceivable that these two activities compete for the end-matured tRNA^His^ that is exported from the nucleus after initial processing events that remove leader and trailer sequences. Here, we used in vitro enzyme assays to investigate the 5′- and 3′-end maturation of eukaryotic cytosolic tRNA^His^ by the two enzymes responsible for these activities. Based on tRNA^His^-dependent kinetic parameters as well as fidelity defects in the activity of Thg1, our results support a scenario in which the CCA-adding enzyme is likely to act prior to Thg1.

We note that the kinetic approaches applied to analysis of each of these enzymes were different, with CCA-adding activity measured under steady-state conditions, while Thg1 was measured using the single-turnover regime. This difference was largely driven by substantially different velocities known to be exhibited by these enzymes, which makes it challenging to perform the long-term courses needed to measure steady-state parameters of slowly-reacting Thg1, and would require highly specialized equipment to measure single-turnover parameters of the rapidly-reacting CCA-adding enzyme. Despite this disparity in approach, the k_cat_/K_M_ value in steady-state and k_max_/K_D_ value in single-turnover are analogous reflections of the “specificity constant” for any particular substrate, and in each case support our conclusions that CCA-adding enzyme is likely to act prior to Thg1 (Table 1 and Table 2). Moreover, while these in vitro reaction rates may not precisely reflect the actual rates in vivo, especially given the use of otherwise unmodified tRNA transcripts for these assays, the trend of faster reaction with the CCA-adding enzyme also agrees well with our prediction that this enzyme is the first player in tRNA^His^ maturation in *S. cerevisiae*.

Taken together, the CCA incorporation assays and Michaelis–Menten kinetics indicate that the 3′-end of wild type tRNA^His^ from *S. cerevisiae* is not affected by the maturation status of the 5′-end (Figure 2, Table 1). The apparent K_M_ values for CCA incorporation by yeast CCA-adding enzyme are in a low µM range, indicating similar affinities to both tRNA variants. Those evaluated apparent K_M_ correspond to previously found K_M_ values for tRNA structures or tRNA-like structures [27,28,29]. In addition, the k_cat_ values do not show a significant difference in tRNA-turnover behavior. Contrary to previous observations that the CCA-adding enzyme prefers a single-stranded discriminator base or even more unpaired nucleotides at the 3′-end [30,31,32], the additional guanosine residue at the 5′-end does not appear to sterically hinder the binding of the 3′-OH group of the discriminator, which serves as the nucleophile for the subsequent CTP incorporation. Consequently, no inhibition of the incorporation of the essential CCA triplet is detectable even in the presence of G-1.

In contrast to the maturation status of the tRNA^His^ 5′-end, the presence of the 3′-terminal CCA sequence has a significant effect on G-1 addition as indicated by our time series experiments and the obtained kinetic parameters under single-turnover conditions, which all favor the +CCA substrate over the ΔCCA versions (Figure 3, Table 2). Moreover, the presence of the 3′-CCA exerts an interesting effect on the productive interaction with the tRNA substrate, evident from the significantly lower fractions of maximal product formation observed in time course assays with substrates that lack CCA (Figure 3C and Figure 4C). Although the precise molecular basis for this effect is not clear from existing structures, the absence of CCA appears to affect the ability of the tRNA to adopt a conformation that is accommodated correctly into the Thg1 active site for catalysis.

The preference of Thg1 for the CCA-containing tRNA^His^ extends beyond kinetics and appears to also be reflected in a modest loss of fidelity that could occur if the enzyme acted prior to the CCA-adding enzyme. Here, we clearly detected the incorporation of A-1 on tRNA^His^ΔCCA (Figure 3 and Figure S2). While this resulting product, tRNA^His^+A-1, might be suitable for the CCA-adding enzyme, since there was little effect of the presence of a G-1 nucleotide on its activity, this could render the tRNA^His^ defective by affecting its fitness for aminoacylation by HisRS. However, in vitro kinetic analysis reveals that *S. cerevisiae* HisRS is only modestly impacted in its ability to aminoacylate tRNA^His^ transcripts containing A-1 instead of the natural G-1 nucleotide [33]. Moreover, several other studies have revealed that the identity of the nucleotide base at position N-1 is not as important as the presence of a single phosphate group on the incorporated N-1 residue [34], and that human tRNA^His^ even contains a relatively minor fraction of U-1-containing tRNA^His^ that is presumably generated by the Watson–Crick-dependent 3′-5′ polymerase activity of the human Thg1 homolog [35]. Nonetheless, it is possible that our in vitro assay conditions underestimate the extent of this fidelity issue since the experimental NTP concentrations employed for Thg1 assays do not match physiological conditions. In fact, the concentrations of ATP (0.1 mM; required for the tRNA 5′-adenylation step) and GTP (1 mM; to be incorporated at the -1 position) are ~30 times less and 2 times more, respectively, in our experiments than previously published physiological concentrations [36]. Taking this into consideration, one can postulate that Thg1 infidelity in terms of A-1 addition might be exacerbated in the presence of higher ATP (and lower GTP) concentrations in the cell, if the enzyme acts on tRNA prior to the action of the CCA-adding enzyme.

In addition to detectable amounts of A-1 addition, multiple nucleotide incorporation was observed on tRNA^His^+CCA A73C, as has been observed previously (Figure 4) [25,37]. Incorporation of multiple G residues, up to G-3, on any tRNA would predictably be detrimental to the stability of the tRNA [38]. From an evolutionary standpoint, it appears to be very advantageous for eukaryotic organisms to encode tRNA^His^ A73 in order for Thg1 to incorporate a single G-1. Notably, this same observation raises interesting questions about the maturation of tRNA^His^ in some Bacteria and Archaea that encode homologs of both Thg1 and CCA-adding enzymes and naturally also encode tRNA^His^ with a C73 discriminator. This issue is further complicated by the fact that the 5′-G-1 and 3′-CCA are frequently genomically encoded in these species where the enzymes may also serve repair functions in vivo [39]. In contrast to Thg1, the CCA-adding enzyme does not show a favored discriminator base in the case of tested tRNA^His^ variants (Figure S1). An explanation for this equal acceptance by the CCA-adding enzyme might be related to the composition of the mitochondrial tRNA^His^ in yeast, which differs dramatically in the sequence within the acceptor stem. Like most bacterial tRNA^His^ sequences, the mt-tRNA^His^ from *S. cerevisiae* carries a genomically encoded G-1 across from C73, alleviating the need for Thg1 activity in mitochondria [40]. Due to this sequence difference between cytosolic and mitochondrial tRNA^His^ in yeast, the CCA adding enzyme has to accept both tRNA substrates and interact just as well with the cytosine at position 73, although it is not favored [27].

In summary, the CCA-adding enzyme has a higher turnover number and does not show preference for either tRNA^His^ substrate, while Thg1 relies on the presence of CCA for binding, rates of incorporation, and fidelity. Hence, these data indicate that although many tRNA maturation steps may occur in a rather random order, nucleotide incorporation at 5′- and 3′-ends of eukaryotic tRNA^His^ likely follows a temporal order with the CCA-adding enzyme incorporating 3′-CCA before Thg1 incorporates a single 5′-G-1.

## 4. Material and Methods

### 4.1. Preparation of Recombinat Enzymes

#### 4.1.1. Yeast CCA-adding Enzyme

As the coding sequence for the CCA-adding enzyme from *Saccharomyces cerevisiae* contains no introns, genomic DNA was used for amplification using specific flanking primers. The PCR product was cloned into the *Xho* I and *Nde* I site of pET28a(+). The yeast CCA-adding enzyme was overexpressed with a C-terminal His-tag in *E. coli* BL21(DE3)cca^−^::cam as described [41] and stored at −80 °C in the presence of 40% glycerol until use. SDS-PAGE electrophoresis followed by staining with Coomassie Brilliant Blue R-250 (BioRad) revealed a >90% purity of the preparation. Protein concentration was measured according to Bradford [42].

#### 4.1.2. Yeast tRNA^His^ Guanylyltransferase (Thg1)

Thg1 was overexpressed in *E. coli* BL21(DE3) pLysS cells and purified as described for N-terminally His_6_-tagged enzymes [16,23]. The protein was dialyzed to exchange into 50% glycerol and stored at −20 °C until use. Thg1 was assessed for >90% purity by visual inspection using standard SDS-PAGE. Concentration of Thg1 was determined by BioRad assay.

### 4.2. Preparation of tRNA Substrates

DNA templates for T7-based in vitro transcription were prepared by overlap extension PCR. Transcription of tRNA^His^ variants (tRNAHisΔG-1, tRNA^His^+G-1, tRNA^His^ΔCCA, tRNA^His^+CCA, tRNAHisΔG-1 A73C, tRNA^His^+G-1 A73C, tRNA^His^ΔCCA A73C, and tRNA^His^+CCA A73C) was carried out as described previously, using flanking ribozyme sequences to generate defined homogeneous 5′- and 3′-ends [43,44]. For internally labeled tRNA^His^, transcription was performed in the presence of α-^32^P-ATP (Hartman Analytics). In vitro transcripts for Thg1-catalyzed G-1 addition were 5′-labeled using γ-^32^P-ATP (Perkin Elmer) and T4 polynucleotide kinase according to the manufacturer’s instructions (NEB).

### 4.3. In Vitro CCA-Addition

Standard CCA-addition and kinetic assays under steady-state conditions were performed as described [27,28]. Kinetic parameters of three independent experiments were analyzed using GraphPadPrism (curve fitting by nonlinear regression). Due to the solubility properties of RNA, no excessive saturation concentrations could be used, and the obtained values represent therefore apparent values, as frequently described for CCA-addition [18,19,20,21,45].

### 4.4. In Vitro G-1 Addition: Phosphate Protection Assay

Activity assays contained <200 fmol 5′-^32^P-tRNA (specific activity 6000 Ci/mmol) in 25 mM HEPES pH 7.5, 10 mM MgCl_2_, 3 mM DTT, 125 mM NaCl, 0.2 mg/ml bovine serum albumin (BSA), 0.1 mM ATP and 1 mM GTP. Enzyme was added to a desired final concentration. Aliquots were removed at time points and quenched with 1 mg/ml RNase A (Ambion) and 50 mM EDTA. Each sample was incubated at 50 °C for 10 min. RNase digested samples were treated with 0.05 U calf intestinal alkaline phosphatase (CIAP) (Invitrogen) and incubated at 37 °C for 1 hour. Reaction products were resolved using thin layer chromatography (TLC) in a 1-propanol:NH_4_OH:H_2_O (55:35:10) solvent system. TLC plates were visualized using a Typhoon (GE Healthcare) and quantified using ImageQuant software (GE Healthcare).

Single-turnover time courses were plotted and fit to a single exponential Equation (1) using Kaleidagraph (Synergy software). P_t_ is the fraction of product formed at each time point and ∆P is maximal amount of product formed for each time course.

(1) $$P_{t}=\Delta P(1-e^{\left(-k_{obs}*t\right)})$$

k_obs_ values obtained with varying concentrations of enzyme were plotted and fit to Equation (2) to yield pseudo first order maximal rate constants (k_max_) and apparent dissociation constant (K_D,app_), which were used to derive the specificity constant k_max_/K_D_,app. Reported errors are least fit squares of the standard deviation derived from the fit using Kaleidagraph [46].

(2) $$k_{obs}=\frac{k_{max}*\left[Thg1\right]}{K_{D,app,tRNA}+\left[Thg1\right]}$$

## Figures and Tables

**Table 1:** Kinetic parameters determined for the CCA-adding enzyme on tRNA^His^ variants carrying the discriminator A73.

Substrate	K_M_ [µM]	k_cat_ [min^−1^]
tRNA^His^∆G-1	1.6 ± 0.6	27.5 ± 3.3
tRNA^His^+G-1	2.9 ± 1.0	25.1 ± 3.3

Data are means ± SD; n = 3

**Table 2:** Kinetic parameters for Thg1 on variant tRNA^His^ substrates.

	tRNA^His^+CCA	tRNA^His^∆CCA	tRNA^His^+CCA A73C	tRNA^His^∆CCA A73C
***k*_obs_** **(min^−1^)**	0.014 ± 0.001	0.0070 ± 0.0004	0.097 ± 0.008	0.013 ± 0.001
**K_D_,_app,tRNA_** **(µM)**	5.8 ± 4.3	>30 *	3.5 ± 1.7	>30 *
***k*_max_/K_D,app_** **(µM^−^^1^min^−^^1^)**	385 ± 45	ND	110 ± 7.7	ND

Data are means ± SD; n = 3; ND: not determined. * saturation was not reached even at the highest possible concentration of purified protein in the assays, leading to the lower estimate for K_D,app_ of 30 µM

## Supplementary Materials

Supplementary materials can be found at https://www.mdpi.com/1422-0067/20/6/1384/s1.

## Abbreviations

aaRS	aminoacyl-tRNA synthetase
DTT	dithiothreitol
EDTA	2,2′,2″,2‴-(Ethane-1,2-diyldinitrilo)tetraacetic acid
HEPES	4-(2-hydroxyethyl)-1-piperazineethanesulfonic acid
HisRS	histidyl-tRNA synthetase
PEI	Polyethylenimine
Thg1	tRNAHis guanylyltransferase
TLC	thin layer chromatography
