# Supplementary material for: A Temporal Order in 5′- and 3′- Processing of Eukaryotic tRNAHis
Source: Int J Mol Sci. 2019 Mar 19;20(6):1384. doi: 10.3390/ijms20061384 (PMC6470698; doi:10.3390/ijms20061384)
Supplement: Supplementary file 1 [file ijms-20-01384-s001.pdf]

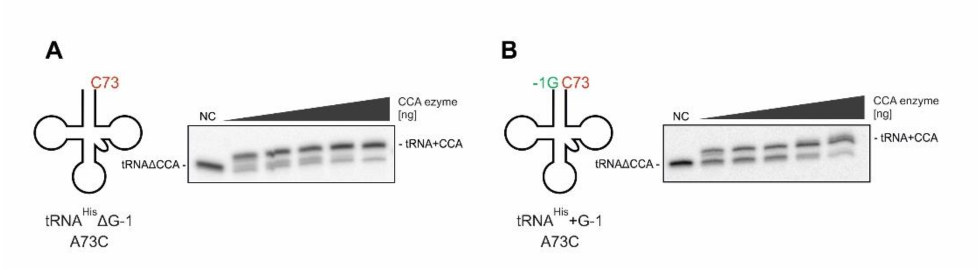

Figure S1: CCA incorporation on radioactively labeled tRNA^{His} variants carrying a C73 discriminator. tRNA^{His} substrates without (tRNA^{His}ΔG-1 A73C; **A**) or with G-1 (tRNA^{His}+G-1 A73C; **B**) are readily accepted for CCA-addition by the yeast CCA-adding enzyme, indicating that the nature and base pair status of the discriminator position in tRNA^{His} has no dramatic effect.

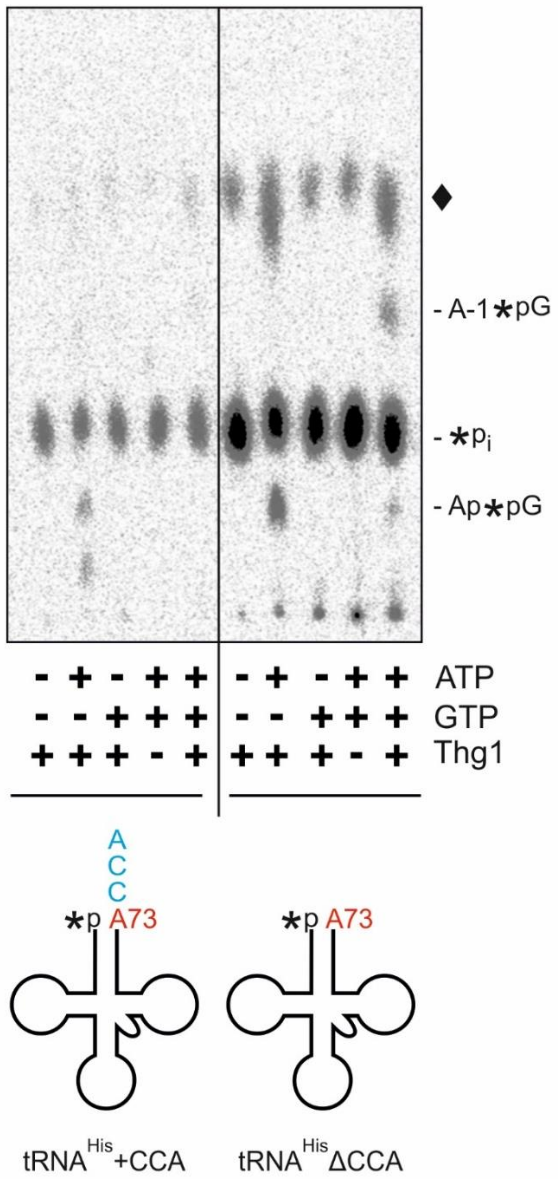

Figure S2: Characterization of A-1 product formation. 5′-end labeled tRNA^{His}+CCA (left) and tRNA^{His}ΔCCA (right) were incubated with saturating amounts of Thg1 (15 μM). The reactions were treated with RNase A/CIAP prior to RNase T1 and resolved by PEI cellulose TLC in 0.5 M sodium formate solution (pH 3.5). When incubated with ATP only, Thg1 catalyzes the first step of the reaction on both substrates (tRNA^{His}+CCA and tRNA^{His}ΔCCA), resulting in the production of 5′-adenylylated tRNA^{His} (represented by the Ap*pG spot indicated on the TLC image). When ATP and GTP are in the reaction, Thg1 readily converts the activated product to G-1 containing tRNA on tRNA^{His}+CCA (left panel), which is visualized as an inorganic phosphate (*P_i) in this assay, since it is sensitive to phosphatase removal after digestion of the labeled tRNA with RNase T1 (which cleaves 3′- to G-nucleotides). When tRNA^{His}ΔCCA (right panel) is tested in the presence of both ATP and GTP, Thg1 converts the activated product to a small fraction of A-1 product. This A-1-containing product is visualized as an A-1*pG dinucleotide that can be resolved from the other RNase T1/CIP cleavage products, as indicated on the TLC image. The non-enzymatic species that is observed even in the absence of added enzyme (lane 4 of each panel) is labeled with ♦ as in previous assays.
